# Anterior STEMI associated with decreased strain in remote cardiac myocardium

**DOI:** 10.1007/s10554-021-02391-0

**Published:** 2021-09-05

**Authors:** Hannah Sjögren, Ulrika Pahlm, Henrik Engblom, David Erlinge, Einar Heiberg, Håkan Arheden, Marcus Carlsson, Ellen Ostenfeld

**Affiliations:** 1 Department of Clinical Sciences Lund, Clinical Physiology, and Skane University Hospital, Lund University, Lund, Sweden; 2 Department of Clinical Sciences Lund, and Anesthesia and Invasive Care, Helsingborg Hospital, Lund University, Lund, Sweden; 3 Department of Clinical Sciences Lund, Cardiology, and Skane University Hospital, Lund University, Lund, Sweden; 4 Department of Clinical Physiology, Skåne University Hospital, 221 85 Lund, Sweden

**Keywords:** Longitudinal strain, ST-elevation infarction, Cardiac magnetic resonance, Feature tracking, Culprit vessel, Infarct size

## Abstract

To assess (1) global longitudinal strain (GLS) by feature tracking cardiac magnetic resonance (CMR) in the sub-acute and chronic phases after ST-elevation infarction (STEMI) and compare to GLS in healthy controls, and (2) the evolution of GLS and regional longitudinal strain (RLS) over time, and their relationship to infarct location and size. Seventy-seven patients from the CHILL-MI-trial (NCT01379261) who underwent CMR 2–6 days and 6 months after STEMI and 27 healthy controls were included for comparison. Steady state free precession (SSFP) long-axis cine images were obtained for GLS and RLS, and late gadolinium enhancement (LGE) images were obtained for infarct size quantifications. GLS was impaired in the sub-acute (− 11.8 ± 3.0%) and chronic phases (− 14.3 ± 2.9%) compared to normal GLS in controls (− 18.4 ± 2.4%; p < 0.001 for both). GLS improved from sub-acute to chronic phase (p < 0.001). GLS was to some extent determined by infarct size (sub-acute: r^2^ = 0.2; chronic: r^2^ = 0.2, p < 0.001). RLS was impaired in all 6 wall-regions in LAD infarctions in both the sub-acute and chronic phase, while LCx and RCA infarctions had preserved RLS in remote myocardium at both time points. Global longitudinal strain is impaired sub-acutely after STEMI and improvement is seen in the chronic phase, although not reaching normal levels. Global longitudinal strain is only moderately determined by infarct size. Regional longitudinal strain is most impaired in the infarcted region, and LAD infarctions have effects on the whole heart. This could explain why LAD infarcts are more serious than the other culprit vessel infarctions and more often cause heart failure.

## Introduction

Survival rates after ST-elevation myocardial infarction (STEMI) have improved substantially over the last decades, however both morbidity and mortality remain high [1–3]. Left ventricular (LV) dysfunction and heart failure are common consequences of STEMI and lead to increased mortality [4, 5]. Thus, there is a need to evaluate LV function with high precision to assess the disease progress and to guide in the use of therapeutic interventions. LV ejection fraction (EF) is the standard marker for systolic function [6], but fails to detect subtle changes in myocardial contractility [7]. Recent studies have suggested global longitudinal strain (GLS) as a measure of dysfunction, especially when EF remains normal [8–10].

Echocardiography is the first-line modality to assess cardiac function and strain [11], and GLS assessed by speckle tracking echocardiography has prognostic implications [8, 12–16]. The acoustic window can be an acquisitional challenge and hence strain analysis done with this technique can be unreliable. Cardiac magnetic resonance (CMR) is the gold standard for volumetric and functional assessment owing to the high precision, accuracy and reproducibility [17]. Feature tracking (FT) enables strain assessment from standard cine CMR images without the need for special sequences [18]. The clinical importance of GLS assessment from FT-CMR has been shown as an independent predictor for major adverse cardiac event [9, 19]. Further, GLS from FT-CMR has added prognostic value to quantifying infarct size (IS) and EF for all-cause mortality [9] and has been presented as a strong and independent predictor of major adverse cardiac events in patients with STEMI [20].

GLS has been studied in the acute phase and at follow up in patients with STEMI using echocardiography [21, 22] and in patients with anterior infarctions using FT-CMR [23]. However, it is unknown how the location of the STEMI, defined by culprit lesion coronary perfusion territory, affect GLS assessed with FT-CMR. Furthermore, it is not known how regional longitudinal strain (RLS) using FT-CMR is affected in the culprit vessel myocardial territory compared to remote territory in the sub-acute phase or how RLS affected in the chronic phase.

We have previously shown that atrioventricular plane displacement is decreased in both the sub-acute and chronic phases after STEMI, resulting in a decreased longitudinal contribution to stroke volume in both phases [24–26]. While longitudinal contribution to stroke volume reflects the volumetric results of the contraction, longitudinal strain measures deformation at a myocardial level.

Therefore, the aims of this study were: (1) to assess GLS with FT-CMR 2–6 days (sub-acute phase) and 6 months (chronic phase) after STEMI compared with healthy controls, and (2) to investigate the evolution of GLS and regional longitudinal strain (RLS) over time, and their relationship to infarct location and size assessed by CMR.

## Methods

### Study population

Patients from the multi-centre cardio-protection trial CHILL-MI (ClinicalTrials.gov NCT01379261) constituted the study population for this study [27]. In short, patients were over 18 years old, presented with chest pain lasting for less than 6 h, had their first STEMI and underwent subsequent percutaneous coronary intervention with successful reperfusion of the occluded vessel. The patients included in this study (n = 77) underwent a CMR examination within 2–6 days after STEMI and a follow-up CMR 6 months later. For comparison, 27 healthy, age-matched controls used in previous studies in our research group, were included [24, 28].

### CMR image acquisition

Patients underwent CMR at one of multiple European centres using two different vendors: Philips Healthcare (Best, The Netherlands) or Siemens AG (Erlangen, Germany). Steady state free precession (SSFP) cine images in short-axis view (base to apex) and in 2-, 3- and 4-chamber long-axis views were acquired. Typical image parameters: 20–30 time frames per cardiac cycle, retrospectively ECG gated, 8 mm slice thickness (no gap) with in-plane resolution 1.5 × 1.5 mm covering the entire left ventricle.

Late gadolinium enhancement (LGE) images were acquired 15–20 min after gadolinium-based contrast agent (0.2 mmol/kg) injection in the corresponding short- and long-axis view as for the SSFP cine imaging. All images were obtained at end-expiratory breath-hold with patients in supine position. Typical image parameters: prospective ECG gating with 8 mm slice thickness (no gap) with in-plane resolution was 1.5 × 1.5 mm. Inversion time was chosen to null remote myocardium [29].

### Image analysis

#### Baseline data

All CMR image analysis was performed using a freely available software for baseline data and infarct size (Segment v1.9 R3084, Medviso, Lund, Sweden) [30] and for strain analysis (Segment v2.2 R6887, Medviso, Lund, Sweden). Delineations of the endo- and epicardium of the LV in short axis cine images in end-diastole and end-systole were made by expert readers (MC, HA, HE) in a core lab setting (Imacor AB, Lund, Sweden) from which LV end-diastolic and end-systolic volumes were computed. LV mass was calculated as epicardial volume subtracted with the endocardial volume and multiplied with the muscle density (1.05 g/ml).

#### Infarct size

**Fig. 1:**
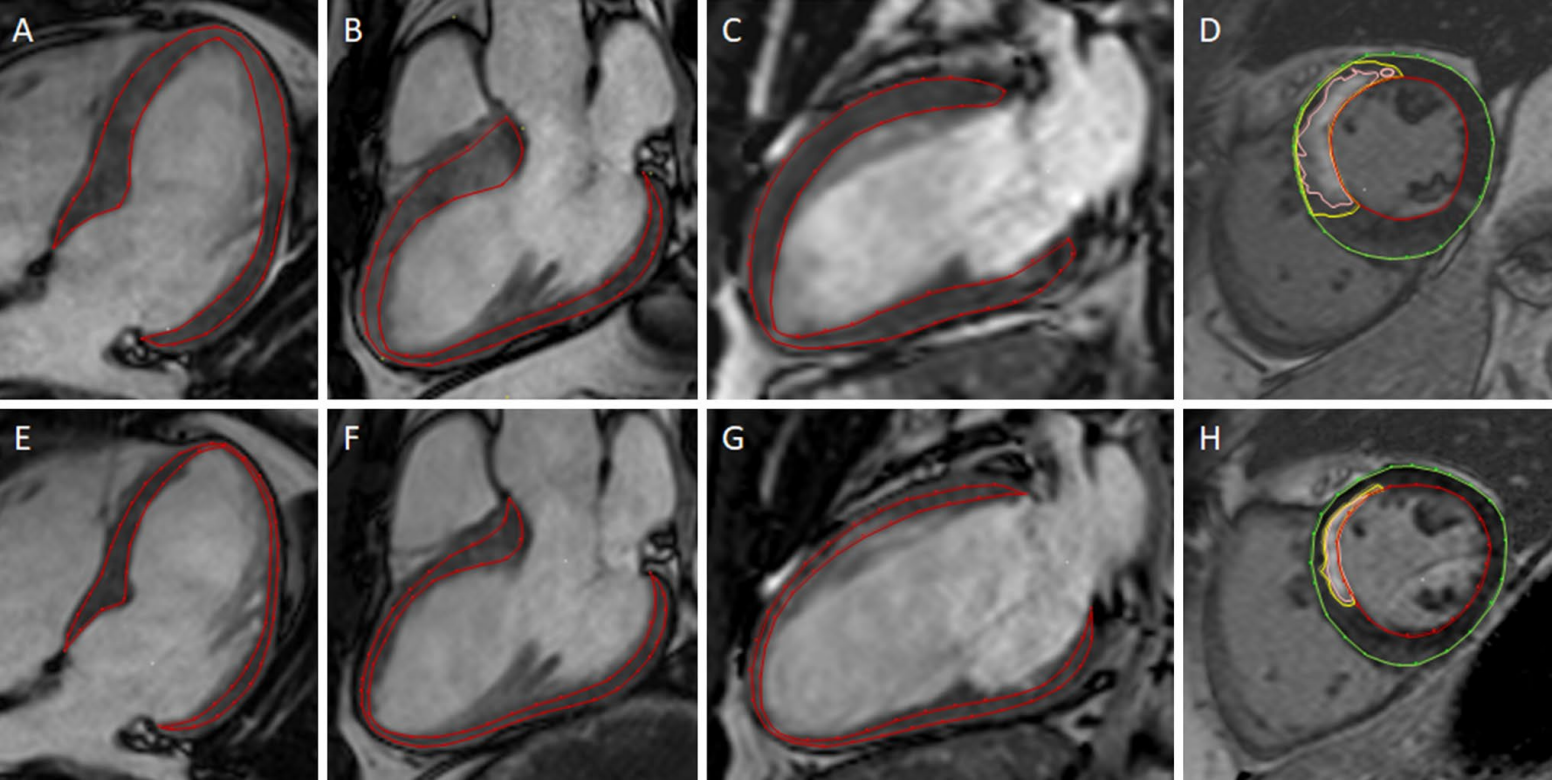
Example of strain and infarct delineations in a patient with STEMI in the LAD in the sub-acute (top row) and chronic (bottom row) phase. **A**–**D** show a patient in the sub-acute phase (2–6 days) after ST-elevation myocardial infarction (STEMI) in the left anterior descending artery (LAD), and **E**–**H** show the same patient in the chronic phase 6 months after the STEMI. The end-diastolic delineations of the left ventricular myocardial borders in red are illustrated in the 4-chamber view (**A**, **E**), 3-chamber view (**B**, **F**) and 2-chamber view (**C**, **G**). Infarct delineation is illustrated in one midventricular short-axis late gadolinium enhancement image (**D**, **H**) with epicardial contour (green), endocardial contour (red), extent infarction (pink) and core of infarction (yellow)

Short-axis LGE images were used to quantify infarct size with a previously validated semi-automatic method using an automatic threshold computation followed by post processing to remove spurious infarct regions and a weighted summation to account for partial volume effects [31], where the endo- and epicardium were manually traced after which a computer algorithm calculated the hyper-enhanced myocardium (MC, HA, HE) (Fig. 1). Manual adjustments were then made when necessary. Infarct size was expressed as percentage of the total LV myocardial volume. Infarct extent was expressed as the proportion of endocardium involved in the infarction in relation to the total LV endocardial surface as previously described [32]. Remote myocardium was defined as absence of infarction assessed by LGE [25, 33]. Infarct transmurality was expressed in percentage and calculated as the amount of infarcted myocardium divided by the myocardial volume, taken over the area of infarction. Amount of infarcted myocardium was also here computed with weighted algorithm [31].

#### Longitudinal Strain

Peak longitudinal strain was analysed both for GLS and RLS in the three long-axis (2-, 3-, and 4-chamber view) cine SSFP images using feature tracking (Fig. 1). Delineations along the epi- and endocardial borders of the myocardium were drawn manually in end-diastole with automated propagation of the myocardial movement throughout the cardiac cycle using a vector-based algorithm [34]. Manual adjustments of the delineations were applied in end-diastole if the automated propagation did not comply properly through systole.

All images were surveyed by one experienced reader (HS) and adjusted by a second expert reader (EO) as adjudicator when needed. If tracking was still inadequate, segments of this view was excluded, and the two remaining views were used for calculations.

Longitudinal strain values were extracted both as an average of the whole LV (GLS) and in 17 LV segments based on American Heart Association’s segmentation model [22]. RLS was compared among patient subgroups divided by STEMI culprit vessel [left anterior descending artery (LAD), left circumflex artery (LCx) and right coronary artery (RCA)].

When assessing RLS, the segments were grouped into six anatomical wall regions: *anterior, anterolateral, inferolateral, inferior, inferoseptal and anteroseptal* wall. Apical segments and the apex (segment 17) were excluded for RLS comparisons as LAD infarctions often has a “wrap around” infarction with larger involvement of apical segments compared to LCx and RCA infarctions.

### Statistics

Continuous data was expressed as mean ± SD. Categorical data was expressed in absolute numbers and proportion in percentage. Student’s t-test and Fisher’s Exact test were used for comparison between all patients and controls. Comparisons between the sub-acute and chronic phase for all patients were made with paired Student’s t-test. Coefficient of determination was calculated from Pearson’s correlation. Subgroup analyses by culprit vessel was—owing to non-normal distribution assessed by histograms—performed with Mann–Whitney U-test for comparison to controls, Wilcoxon Signed Ranks Test for comparison between the sub-acute and chronic phase and Kruskal–Wallis for multiple comparisons with Bonferroni’s correction for multiple tests. Results with a two-sided p-value < 0.05 were considered statistically significant.

Statistical analyses were performed using SPSS (IBM Corp. Released 2017. IBM SPSS Statistics for Macintosh, Version 25.0. Armonk, NY: IBM Corp.) and GraphPad Prism (version 8.00 for Macintosh, GraphPad Software, San Diego, California USA). Radar charts were made in Microsoft Excel 2015.

## Results

**Fig. 2:**
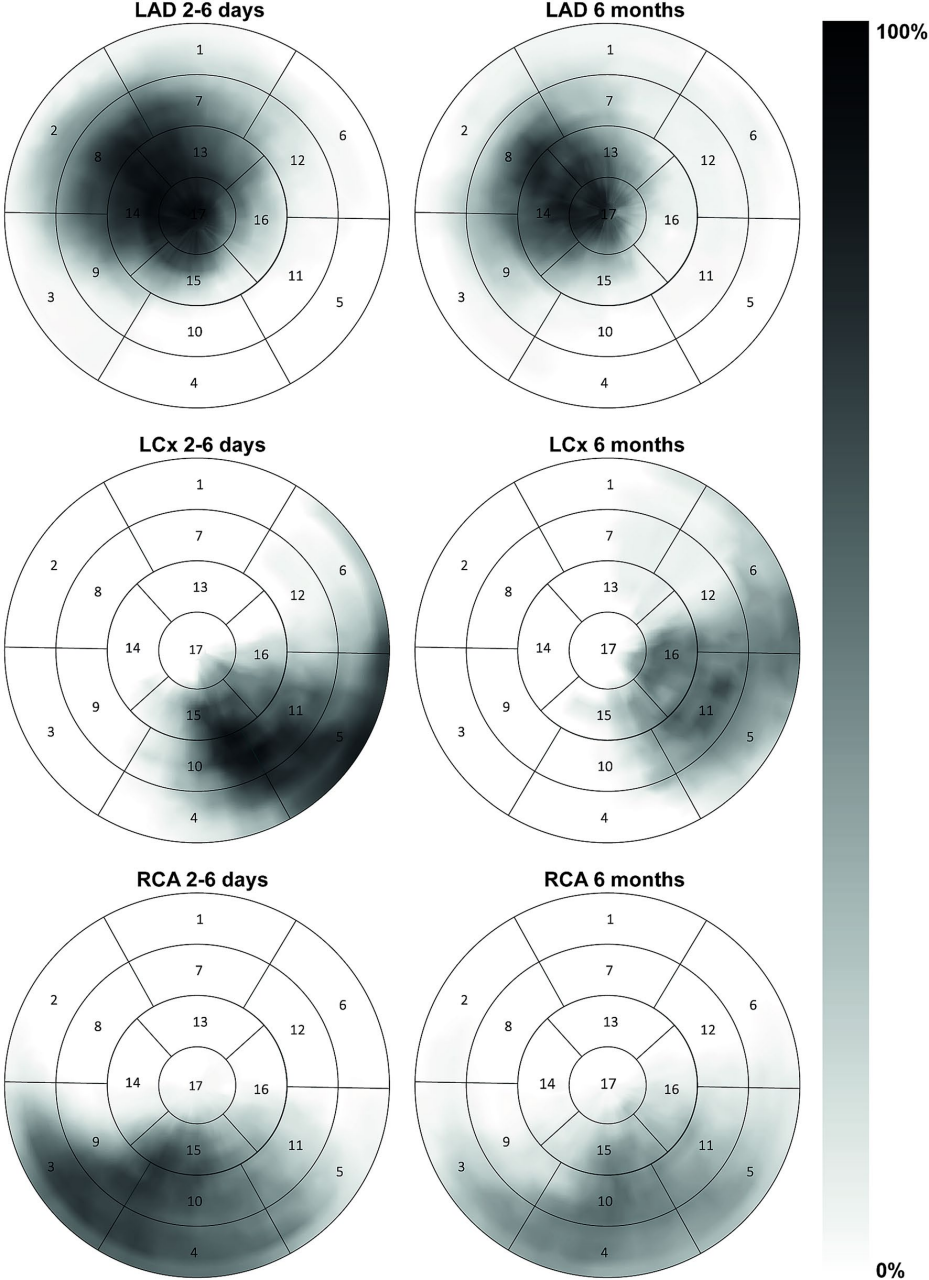
Bullseye plots over distribution of infarction after STEMI. Polar plots of the distribution of infarction in the sub-acute and chronic phase after STEMI in the left anterior descending artery (LAD), left circumflex artery (LCx) and right coronary artery (RCA). The grayscale intensity represents the average amount of infarction, expressed in percentage, as shown on the bar to the right with white representing 0% and black 100% infarction

Seventy-seven patients (58.2 ± 10.2 years) and 27 age matched controls (60.7 ± 10.7 years) were included. At admission, none had prior myocardial infarction, percutaneous coronary intervention, coronary artery by-pass graft, or congested heart failure. One (1%) had prior stroke, 8 (10%) had known diabetes mellitus, 10 (13%) had medication for hyperlipidaemia, and 20 (26%) had antihypertensive medication. Twenty-seven (35%) had other significant lesions than the culprit lesion. Data on medical therapy at follow-up was not available in this study. Patients were divided into groups according to culprit vessel (LAD n = 27, LCx n = 10 and RCA n = 39). Figure 2 demonstrates the distribution of infarction on a group level for each culprit vessel in the sub-acute and chronic phase after STEMI. Subject characteristics are presented in Table 1. Patients and controls did not differ in age, but the patient group had a higher proportion of men than the control group. Weight and end-systolic volumes were higher and EF lower in patients than in controls. Of note, heart rate, LV mass and SV were altered in the sub-acute phase but normalised to the chronic phase to levels that are *en par* with the healthy controls.

**Table 1:** Subject characteristics

	Patients		Controls
	2–6 days	6 months	
N	77	77	27
Women/men	8/69 (10/90%)*	8/69 (10/90%)*	8/19 (30/70%)
Age (years)	58.2 ± 10.2		60.7 ± 10.7
Weight (kg)	83.0 ± 10.9*	82.2 ± 10.9*	76.6 ± 12.3
HR (bpm)	68.6 ± 11.2***	61.4 ± 9.5^###^	61.0 ± 7.6
LVM (g)	126.7 ± 26.3*	111.8 ± 25.8^###^	113.2 ± 27.8
EDV (ml)	178.6 ± 36.5	188.9 ± 42.1*^###^	167.0 ± 33.2
ESV (ml)	93.5 ± 30.8***	92.2 ± 33.8***	68.9 ± 19.0
SV (ml)	85.1 ± 16.5***	96.6 ± 21.6^###^	98.1 ± 18.2
EF (%)	48.5 ± 8.3***	52.0 ± 9.4***^###^	59.1 ± 5.2

Data expressed as mean ± standard deviation or in absolute numbers and (percentage)

*LVM* left ventricular mass, *LVEDV* left ventricle end-diastolic volume, *LVESV* left ventricle end-systolic volume, SV stroke volume, *EF* ejection fraction

*p < 0.05 compared to controls

**p < 0.01 compared to controls

***p < 0.001 compared to controls

^#^p < 0.05 compared to 2–6 days

^##^p < 0.01 compared to 2–6 days

^###^p < 0.001 compared to 2–6 days

### Global longitudinal strain (GLS)

**Fig. 3:**
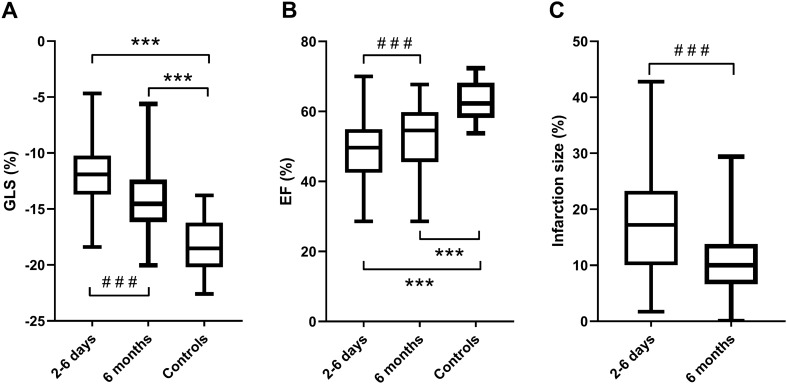
Global longitudinal strain, ejection fraction and infarction size. Boxes (median and 25th and 75th percentile) with whiskers (min to max). **A** Global longitudinal strain (GLS) for patients at the sub-acute and chronic phase as well as healthy controls. **B** Ejection fraction (EF) for patients at the sub-acute and chronic phase as well as healthy controls. **C** Patients’ infarction size at the sub-acute and chronic phase. Three patients had missing infarct size data at six months, why paired comparisons of IS% was only performed with 74 patients. ***p < 0.001 compared to controls. ^###^p < 0.001 compared to 2–6 days

Four of 2172 segments were excluded owing to inadequate tracking. Table 2 shows GLS and IS for all patients and patients divided by subgroups as well as GLS for controls. Figure 3 shows the distribution and comparison between groups of IS and GLS. Infarct size was significantly larger within the first week of STEMI than six months after (p < 0.001).

**Table 2:** Global longitudinal strain and infarction size

	GLS (%)	IS (%)^a^
All patients		
2–6 days	− 11.8 ± 3.0***	17.4 ± 9.0
6 months	− 14.3 ± 2.9***^###^	10.3 ± 5.8^###^
LAD infarction (n = 28)		
2–6 days	− 9.2 ± 2.6***	23.6 ± 8.4
6 months	− 12.8 ± 2.9***^###^	13.8 ± 6.3^###^
LCx infarction (n = 10)		
2–6 days	− 13.3 ± 1.6***	13.1 ± 5.1
6 months	− 15.3 ± 2.5**^##^	7.3 ± 3.6^##^
RCA infarction (n = 39)		
2–6 days	− 13.3 ± 2.3***	14.1 ± 7.6
6 months	− 15.0 ± 2.7***^###^	8.7 ± 4.8^###^
Controls (n = 27)	− 18.4 ± 2.4	

Table of infarct size (IS) global longitudinal strain (GLS) for all patients, patients divided into subgroups by culprit vessel and controls. Data expressed as mean ± standard deviation

*LAD* Left anterior descending artery, *LCx* Left circumflex artery, RCA Right coronary artery

**p < 0.01 compared to controls

***p < 0.001 compared to controls

^##^p < 0.01 compared to 2–6 days

^###^p < 0.001 compared to 2–6 days

^a^Three patients had missing infarct size data at six months, why paired comparisons of IS% was only performed with 74 patients

GLS was reduced both in the sub-acute and chronic phases compared to controls (p < 0.001 for both). GLS and EF improved from the sub-acute to the chronic phase (p < 0.001) (Fig. 3).

**Fig. 4:**
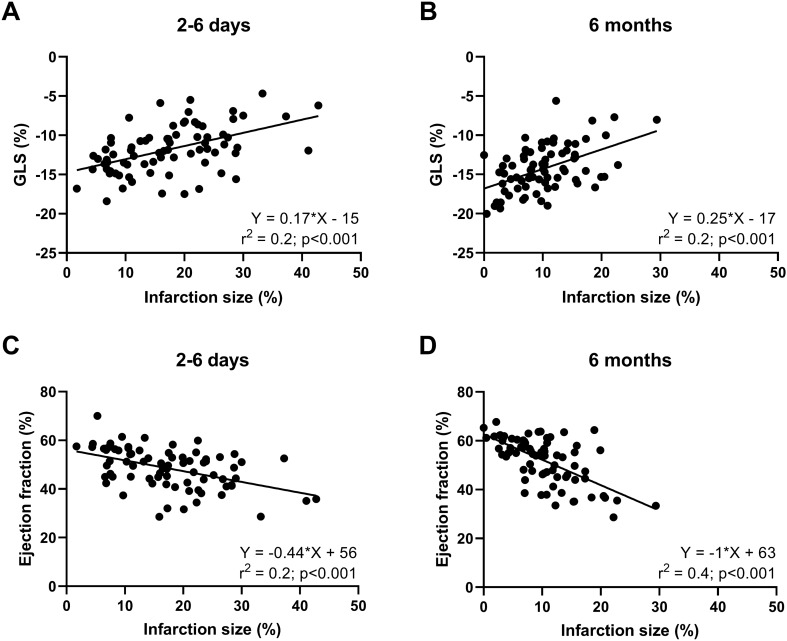
Scatter plot between infarct size, global longitudinal strain and ejection fraction. Scatter plots with linear regression best fit line and coefficient of determination (r^2^) for patients’ infarct size vs. global longitudinal strain (GLS) (**A**, **B**) and ejection fraction (**C**, **D**). **A** and **C** shows the sub-acute phase and B and D the chronic phase

IS and GLS had a modest, but significant coefficient of determination in both the sub-acute and chronic phases after STEMI as well as between IS and EF (Fig. 4). Likewise, there was a significant coefficient of determination between endocardial extent and transmurality of infarction and GLS in chronic phase (r^2^ = 0.2, p < 0.001 for both) after STEMI and between endocardial extent and transmurality of infarction and EF in both phases (extent: sub-acute: r^2^ = 0.3 and chronic: r^2^ = 0.3, p < 0.001 for both; and transmurality: sub-acute: r^2^ = 0.1, p = 0.03 and chronic: r^2^ = 0.2, p < 0.001). There was, however, no significant coefficient of determination between endocardial extent or transmurality of infarction and GLS in the sub-acute phase (extent: r^2^ = 0.01, p = 0.3 and transmurality: r^2^ < 0.001, p = 1.0; respectively).

There was no difference in GLS in the sub-acute phase between patients with or without diabetes (p = 0.4), with or without antihypertensive medication (p = 0.08), or with or without lipid lowering medication (p = 0.2) at admission. Patients with other significant lesions than culprit lesion had lower GLS than those with only one lesion in the sub-acute phase (− 10.8% vs − 12.3%, p = 0.04), while there was no difference in GLS the chronic phase between the groups (− 14.2% vs − 14.3%, p = 0.7).

### Regional longitudinal strain (RLS)

**Fig. 5:**
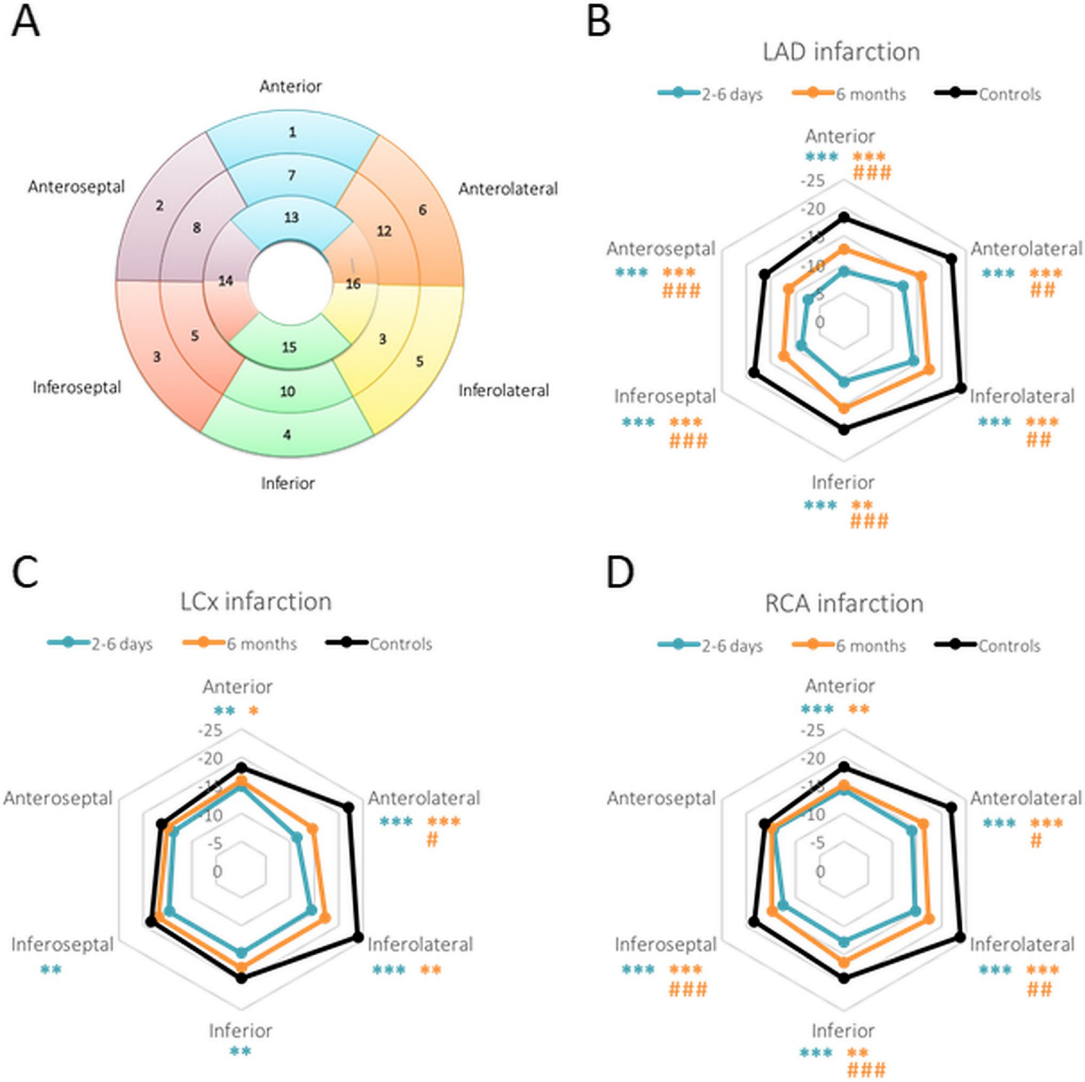
Regional longitudinal strain by myocardial wall regions. Radar charts of longitudinal regional strain divided into 6 walls according to (**A**). Note that the apical cap (segment 17) is excluded and that segments 14 and 16 are both shared by two wall regions. **B**–**D** Chart axis (light gray) show strain, and data points show the mean strain of the segments included in the region. **B** Patients with STEMI in the left anterior descending artery (LAD); **C** Patients with STEMI in the left circumflex artery (LCx); **D** Patients with STEMI in the right coronary artery (RCA). Black represents strain values of healthy controls, blue represents strain values in STEMI patients at 2–6 days, and orange represents strain values in patients at 6 months follow up. *p < 0.05, **p < 0.01, ***p < 0.001 compared to controls. ^#^p < 0.05, ^##^p < 0.01, ^###^p < 0.001 compared to 2–6 days

In patients with LAD infarction, RLS was impaired in all six wall regions both in the sub-acute and chronic phases after STEMI compared to controls, with a significant improvement in the chronic phase in all regions (Table 3, Fig. 5). Patients with LCx and RCA infarctions had impaired RLS in all regions except the anteroseptal wall in the sub-acute phase. In the chronic phase, LCx patients had improved RLS anterolaterally and showed no difference to controls in the inferior and inferoseptal walls. Patients with RCA infarctions also had partial recovery in the anterolateral, inferolateral, inferior and inferoseptal walls from the sub-acute to the chronic phase.

**Table 3:** Regional longitudinal strain by myocardial wall regions

	Anterior	Anterolateral	Inferolateral	Inferior	Inferoseptal	Anteroseptal
LAD infarction (n = 28)						
2–6 days	− 8.8 ± 2.5***	− 12.2 ± 4.6***	− 14.2 ± 5.4^***^	− 10.8 ± 4.0***	− 8.7 ± 3.2***	− 7.4 ± 2.9***
6 months	− 12.7 ± 2.5***^###^	− 15.8 ± 3.3***^##^	− 17.4 ± 5.0***^##^	− 15.5 ± 4.1**^###^	− 12.3 ± 3.9***^###^	− 11.4 ± 3.6***^###^
LCx infarction (n = 10)						
2–6 days	− 14.9 ± 1.7**	− 11.5 ± 3.4***	− 14.4 ± 3.9***	− 14.8 ± 3.8**	− 14.7 ± 2.5^**^	− 13.8 ± 2.9
6 months	− 15.9 ± 2.4*	− 14.7 ± 4.7***^#^	− 17.2 ± 5.2**	− 17.4 ± 4.0	− 16.9 ± 3.5	− 15.1 ± 2.3
RCA infarction (n = 39)						
2–6 days	− 14.3 ± 2.9***	− 14.0 ± 4.3***	− 14.6 ± 4.2***	− 12.8 ± 3.6***	− 12.5 ± 2.9***	− 14.4 ± 3.4
6 months	− 15.1 ± 4.0**	− 16.4 ± 4.1***^#^	− 17.4 ± 4.4***^##^	− 16.4 ± 3.8**^###^	− 14.8 ± 3.9***^###^	− 14.9 ± 3.1
Controls (n = 27)	− 18.3 ± 3.3	− 22.1 ± 4.2	− 23.9 ± 4.4	− 19.3 ± 4.0	− 18.4 ± 2.7	− 16.2 ± 3.4

Data expressed as mean ± standard deviation

*LAD* Left anterior descending artery, *LCx* Left circumflex artery, *RCA* Right coronary artery

*p < 0.05

**p < 0.01

***p < 0.001 compared to controls

^#^p < 0.05

^#^p < 0.01

^###^p < 0.001 compared to 2–6 days

In the sub-acute phase, patients with LAD infarction had lower RLS in the anterior, anteroseptal and inferoseptal walls than patients with RCA or LCx infarction (p < 0.001 for all), while there was no difference between RCA and LCx infarctions in these areas (p = 1.0; p = 1.0 and p = 0.2, respectively). In the inferior wall region, patients with LAD infarctions had worse RLS than patients with LCx infarctions (p = 0.02) and no difference was seen between patients with LAD and RCA infarctions (p = 0.1) or between RCA and LCx infarctions (p = 0.5). In the anterolateral and inferolateral wall, RLS did not differ between site of infarction (p = 0.2 and 1.0, respectively).

In the chronic phase, patients with LAD infarctions still had lower RLS than both patients with LCx and RCA infarctions in the anterior (p = 0.02 and 0.01, respectively) and anteroseptal walls (p = 0.01 and p = 0.001, respectively). In the inferoseptal wall, RLS was worse after infarction in the LAD than in the LCx (p = 0.007) but no significant difference remained compared to RCA infarctions (p = 0.08). No difference was seen in RLS between culprit vessels in the anterolateral (p = 0.7), inferolateral (p = 1.0) or inferior walls (p = 0.6).

## Discussion

The main result of this study was that left ventricular GLS was impaired to a greater degree after a STEMI in the LAD territory compared to LCx and RCA territory. This may in part be explained by the larger IS for the LAD-infarction. In LAD-infarction, RLS is similarly impaired in infarcted and remote myocardium, even in the chronic phase. In contrast, LCx and RCA infarction show larger impairment of RLS in the infarcted territory compared to remote. GLS is, as expected, impaired in the sub-acute phase after STEMI compared to healthy controls, and ameliorates in the first six months, but not to the level of the controls. Further, GLS was moderately associated to IS in both the sub-acute and chronic phase, while GLS was only associated with endocardial extent of infarction in the chronic phase, and not in the sub-acute phase.

### Global longitudinal strain (GLS)

GLS from FT-CMR within the first week after admission has been shown to be a strong and independent predictor of major adverse cardiac events in patients with STEMI [20]. Our findings of improved GLS at 6 months are in concordance with those of Podlesnikar et al. [23] who measured GLS with feature tracking CMR within one week and six months after anterior STEMI and found improvement after six months. Our findings are also aligned with previous studies using speckle tracking echocardiography [21, 22]. The study by Podlesnikar et al. [23] focused on GLS after anterior STEMI as a way of investigating the effect of metoprolol and did not assess IS. Our study included STEMI in all three coronary arteries and investigated RLS why it adds new knowledge about the longitudinal remodeling over time in relations to infarct location, size, and degree. Of note, our results show that LAD infarctions affect the whole left ventricle. This could explain why LAD infarcts are more detrimental than LCx and RCA infarctions and cause more heart failure [20].

### Regional longitudinal strain (RLS)

The evenly distributed decrease in RLS in patients with LAD infarctions could have many potential reasons and underlying mechanisms. First, it could be due to a larger IS seen in the patients with LAD compared to patients with LCx and RCA infarctions. On the other hand, we found only a modest association of GLS and IS, why IS alone cannot explain the decreased GLS and RLS. Secondly, longitudinal strain is preload-dependent and as post-infarction remodeling is altering the preload [13], IS could indirectly through increased preload be an alternative explanation to the lower regional values in the LAD infarctions. A third explanation could be an increase of plasma catecholamine levels to compensate for the greater loss of myocardial function in LAD infarctions compared to non-LAD. An increase in cardiac sympathetic nerve activity and impaired parasympathetic function could induce a deterioration of myocardial function during the ischemic process [35, 36]. The latter could be supported by a study by Baron et al., in which patients with acute infarction prescribed beta-adrenergic blockers at discharge had better improvement in GLS from 1 year after infarction than those without [13]. However, in a study by Karlsberg et al., there was no difference in plasma catecholamine concentrations between acute anterior and inferior infarction [37]. Instead the sympathoadrenal activation seemed to be related to the extent of the acute myocardial damage [37]. In our study, infarct extent and transmurality was not associated to GLS in the sub-acute phase, which means GLS is associated to IS, but not infarct extent or transmurality in this phase. Our study is in concordance with a CMR study by Li et al. showing an association between infarct size and global strain [38]. While they found global radial, circumferential and longitudinal strain differed between patients with transmurality above or below 50% in the anterior wall myocardial infarctions, they did not find any difference in these global strain values in the non-anterior wall myocardial infarctions between the dichotomised transmurality groups. Their study differs from ours by comprising a mix of patients with acute and previous myocardial infarctions, while our study included patients with STEMI only. Furthermore, we assessed infarct size, extent and transmurality as continuous data in both the sub-acute and chronic phase, thereby presenting the evolution of both GLS and RLS over time after STEMI. Moreover, Li et al*.* used a standard deviation method for infarct assessment. Simple thresholds have shown varying results and we used a weighted intensity algorithm validated in patients and experimentally with low bias of infarct quantification [31].

The results from RLS are in concordance to our findings of altered regional atrioventricular plane displacement [25]. The results that impaired regional function is poorly or moderately correlated to site infarction is also in concordance with Pahlm et al. when studying wall motion in an animal setting pre and post infarction acutely [39]. Furthermore, the RLS pattern of LAD, LCx and RCA infarctions in our study aligns to some extent with the results from speckle tracking echocardiography by Biering-Sørensen et al. [36]. They also found generally reduced strain values in all six regions in patients with LAD infarction. However, they found significantly lower strain values in the culprit lesion perfusion territory of LAD, LCx and RCA, while we did not find significant difference in inferior and lateral RLS when comparing culprit lesion perfusion territory. An explanation in difference could be the time from infarction to examination. In the echocardiographic study, patients were examined 1–3 days after the STEMI, and in our study, patients were examined 2–6 days and 6 months after STEMI. A potential stunning of the myocardium could have been present in an acute phase, while it may have—at least partially—been revoked within a week. Of note, an impaired RLS in anterior septal and inferior myocardial walls, irrespective of culprit lesion perfusion territory, has been shown to have prognostic value when using echocardiography [36, 40] but the prognostic value of RLS from feature tracking CMR remains to be investigated.

## Limitations

There were some limitations to our study. First, feature tracking CMR strain is still under development and normal values are not generally applicable for all tracking algorithms. Strain values can differ among the different vendors [41], and therefore data is not interchangeable when using different software. However, we used the same software for all strain assessment why comparison among our groups should be considered valid. Secondly, in the images from the sub-acute phase the tracking algorithm sometimes errs in tracking segments with much edema even if manual adjustments were applied. However, if insufficient tracking persisted despite adjustment, the segments/image/walls were excluded from the analysis. Thirdly, The LCx subgroup was very small with only 10 patients, which must be regarded when making conclusions about strain and IS for this subgroup. Fourth, the effect of medical therapies on GLS over time might be a potential confounder. However, data on medical therapy was not available in this study. Fifth, previous studies have emphasised the clinical usefulness and prognostic relevance of remote tissue characterization by CMR mapping post-STEMI [42]. T1 mapping or outcome were not obtained in the present study. Sixth, in this multi-centre study, cine images were acquired with 20–30 time frames according to local standards. A low frame rate could potentially affect the strain values. In a study by Backhaus et al., GLS was lower when the temporal resolution was 20 time frames compared to 30, 40 and 50 times frames in 5 mm and 10 mm slices [43]. But they found no difference in GLS regarding temporal resolution when using 8 mm slice thickness, such as we did in our study.

## Conclusion

Global longitudinal strain is impaired sub-acutely after STEMI and improvement is seen in the chronic phase, although not reaching normal levels. Global longitudinal strain is only moderately depending on infarct size, indicating that other factors are important for ventricular function after STEMI. Even though regional longitudinal strain is most impaired in the infarcted region, remote regions can be significantly impaired in LAD infarctions, making the identification of culprit vessel from analysis of regional longitudinal strain difficult. Moreover, as LAD infarctions affect the whole heart, this could explain why LAD infarcts are more detrimental than the other culprit vessel infarctions and more often cause heart failure.

## Data Availability

All relevant data are within the manuscript.

## Abbreviations

CMR: Cardiac resonance imaging
EF: Ejection fraction
GLS: Global longitudinal strain
LAD: Left anterior descending artery
LC_x_: Left circumflex artery
LGE: Late gadolinium enhancement
LS: Longitudinal strain
LV: Left ventricular
RCA: Right coronary artery
RLS: Regional longitudinal strain
SSFP: Steady state free precession
STEMI: ST-elevation infarction
SV: Stroke volume
